# Salutatory

**Published:** 1911-07

**Authors:** 


### EDITORIAL DEPARTMENT.

### SALUTATORY.

With this issue the TEXAS MEDICAL JOURNAL begins its 27th year of continuous service under one,—the original management. In entering upon the 27th round its editor, owner and founder salutes its thousands of friends all over this fair and beautiful land, and thanks them for their steadfast loyalty and support, moral and financial. And although the frosts of many winters have whitened the "locks that once were like the raven, John," and wrinkled is that "bonny brow" that once was "brent," he starts on the 27th voyage encouraged by increasing popularity and patronage, with undiminished zeal in the cause of sanitation and legitimate medicine, determined, with health and strength, to maintain the standard and policy which for more than a quarter century has given the "Red Back" its unique place in medical journalism and made it the popular favorite. Selah!*

*It is just too hot to write any "able editorials" this month. I'm going camping, to angle for the nimble black bass, and you'll hear from me next month.

